# Chemicals, Nutrition, and Autism Spectrum Disorder: A Mini-Review

**DOI:** 10.3389/fnins.2016.00174

**Published:** 2016-04-20

**Authors:** Takeo Fujiwara, Naho Morisaki, Yukiko Honda, Makiko Sampei, Yukako Tani

**Affiliations:** ^1^Department of Social Medicine, National Research Institute for Child Health and Development, Okura, Setagaya-ku, Tokyo, Japan; ^2^Department of Global Health Promotion, Tokyo Medical and Dental UniversityTokyo, Japan; ^3^Global Cooperation Institute for Sustainable Cities, Yokohama City UniversityYokohama, Japan; ^4^Department of Health Education and Health Sociology, School of Public Health, The University of TokyoTokyo, Japan

**Keywords:** autism spectrum disorder, air pollution, chemicals, pesticide, fatty acid, micronutrients, heavy metal, environment

## Abstract

The rapid increase of the prevalence of autism spectrum disorder (ASD) suggests that exposure to chemicals may impact the development of ASD. Therefore, we reviewed literature on the following chemicals, nutrient to investigate their association with ASD: (1) smoke/tobacco, (2) alcohol, (3) air pollution, (4) pesticides, (5) endocrine-disrupting chemicals, (6) heavy metals, (7) micronutrients, (8) fatty acid, and (9) parental obesity as a proxy of accumulation of specific chemicals or nutritional status. Several chemical exposures such as air pollution (e.g., particular matter 2.5), pesticides, bisphenol A, phthalates, mercury, and nutrition deficiency such as folic acid, vitamin D, or fatty acid may possibly be associated with an increased risk of ASD, whereas other traditional risk factors such as smoking/tobacco, alcohol, or polychlorinated biphenyls are less likely to be associated with ASD. Further research is needed to accumulate evidence on the association between chemical exposure and nutrient deficiencies and ASD in various doses and populations.

## Introduction

Autism spectrum disorder (ASD) is a developmental disorder typified by impaired communication and social skills (Grabrucker, 2012). A recent increase in cases of ASD from 4–5 of 10,000 persons in 1966 to 100 cases of 10,000 persons currently (Fombonne, 2009) may not solely be explained by genetic factors (Abrahams and Geschwind, 2010). Thus, it needs to be determined whether environmental factors play a role in the onset of ASD (Grabrucker, 2012), and a recent study using twin samples reported that around 50% of cases of ASD can be explained by environmental factors (Hallmayer et al., 2011).

In the present mini-review, we report several relatively new studies that have evaluated the association between ASD and environmental factors by focusing on chemical or nutritional exposures because these are modifiable factors. These exposures included smoking/tobacco, alcohol, air pollution, pesticides, endocrine-disrupting chemicals, heavy metals, micronutrients, and fatty acid. Parental obesity was also included as an exposure because maternal obesity can be an indicator of exposure to chemicals or nutrition.

## Smoke or tobacco

Although not consistent, most recent population-based studies have suggested that maternal smoking during pregnancy is not directly associated with ASD after adjusting for socioeconomic status (Burstyn et al., 2010; Kalkbrenner et al., 2012; Lee et al., 2012; Tran et al., 2013). For example, Lee et al. (2012) performed a population-based nested case-control study of 3958 cases of ASD and 38,983 controls in a longitudinal register-based study consisting of individuals aged 4–17 years, and found that maternal smoking during 8–12 weeks of gestation was significantly associated with an increased odds of high-functioning autism in an unadjusted model (odds ratio [OR] = 1.22, 95% confidence interval [CI]: 1.09, 1.36); however, this finding was no longer statistically significant after adjusting for parental socioeconomic status. Additionally, Tran et al. (2013) conducted a population-based nested case-control study comprising 16,185 samples, including 4020 cases of ASD, based on the Finnish National Birth Cohort. Maternal smoking during all pregnancies was not associated with the offsprings' ASD status after adjusting for confounding factors.

Considering that these studies were conducted mostly among Caucasians, the impact of smoking on the development of ASD may differ by race. Zhang et al. (2010) conducted a case-control study using 190 Han children aged 3–21 years with and without autism in China, and found that maternal second-hand smoke exposure during pregnancy, was significantly associated with autism (OR = 3.53, 95% CI: 1.30, 9.56), suggesting that maternal smoking may be associated with ASD among Asians.

## Alcohol

Few studies have evaluated the impact of maternal alcohol use on the onset of ASD among offspring. Two population-based nested control studies in North European countries reported that maternal alcohol intake during pregnancy was not associated with ASD (Daniels et al., 2008; Eliasen et al., 2010).

## Air pollution

In the last decade, literature on the effect of air pollution exposure during pregnancy on the risk of ASD has grown immensely. Although a large study using direct person-based air sampling is needed, the analytical models used to calculate residence-based effects have become increasingly complex to correctly estimate exposure during a specific time. Regardless of this change in measuring the effect of exposures, most studies have shown a positive association between air pollution exposure and ASD (Suades-Gonzalez et al., 2015). Recent findings support that exposure to particulate matter (PM) < 2.5 μm in diameter (PM_2.5_) during the third trimester causes the most detrimental effect on the development of ASD (Kalkbrenner et al., 2015; Raz et al., 2015; Talbott et al., 2015; Weisskopf et al., 2015).

One of the earlier studies that measured air pollution during pregnancy was conducted in 2011 using the distance of one's residence to major roadways as its proxy. Comparing 304 cases of ASD and 259 controls, Volk et al. (2011) reported that mothers of children with ASD were more likely to have lived near a freeway during their third trimester (OR 2.22, CI: 1.16, 4.42) or at the time of delivery (OR 1.86, CI: 1.04, 4.42). This study raised another research question: which type of air pollution has the most effect on the onset of ASD? Evidence of the effect of ozone or nitro-oxides on ASD has been inconclusive (Becerra et al., 2013; Gong T. et al., 2014; Guxens et al., 2016), but maternal exposure to small particles such as diesel PM (Windham et al., 2006; Roberts et al., 2013), PM 2.5 (Becerra et al., 2013; Volk et al., 2013; Raz et al., 2015; Talbott et al., 2015), and PM < 10 μm in diameter (PM_10_) (Volk et al., 2013; Kalkbrenner et al., 2015) has been most consistently reported with an increased risk of ASD. Several case-control studies have investigated the effect of individual hazardous air pollutants (HAP) such as metals and volatile organics on ASD (Windham et al., 2006; Kalkbrenner et al., 2010; Roberts et al., 2013; von Ehrenstein et al., 2014) with all studies conducted in the United States showing that cases of ASD have an elevated exposure of HAP by 1.3–2.0 times. However, two reports from European countries (Guxens et al., 2016) and a separate twin study in Sweden (Gong T. et al., 2014) showed no association of maternal exposure to air pollution and ASD.

Most studies about the effect of air pollution on ASD have been prone to residual confounders such as a low socioeconomic status, which is related to both worse living environments and an increased risk of ASD (Bell and Ebisu, 2012; Shmool et al., 2014).

## Pesticides

Evidence from previous studies has suggested a strong relationship between pesticide exposure and ASD. Despite the quick turnover in commercial product names, organophosphates (OP) and organochlorines (OC) are still in use despite their neurotoxicity (Kalkbrenner et al., 2014). The association between ASD and pesticides has been observed across studies that measured exposures from residential exposure to agricultural drift (Roberts et al., 2007; Roberts and English, 2013), administered questionnaires on the use of insecticides (Keil et al., 2014), and assessed bio-specimens to detect metabolites (Rauh et al., 2006; Eskenazi et al., 2007; Cheslack-Postava et al., 2013) and numerous pesticides, including but not limited to OC (Roberts et al., 2007; Cheslack-Postava et al., 2013; Roberts and English, 2013; Braun et al., 2014) and OP (Rauh et al., 2006; Eskenazi et al., 2007; Shelton et al., 2014) pesticides.

Shelton et al. (2014) compared 486 cases of ASD and 316 controls, and found an association with OP exposure and ASD, which strengthened later in pregnancy for mothers living within 1.75 km from the agricultural use of OP during their third trimester. They also found increased exposure to pyrethroids in patients with ASD. Eskenazi et al. (2007) and Rauh et al. (2006) reported that cases of ASD had higher OP metabolites during early- to mid-pregnancy. Other case-control studies reported that exposure to imidacloprid through the consistent use of flea/tick pet treatment throughout pregnancy period was associated with ASD (Kalkbrenner et al., 2014; Keil et al., 2014).

## Endocrine-disrupting chemicals

Although, polychlorinated biphenyl (PCB) and several dioxins such as tetrachlorodibenzodioxin were banned by the Stockholm Convention in 2001, they are still detected in humans due to their long half-life in the environment, as well as the consumption of predatory fish in which such chemicals tend to accumulate. Other chemicals are still used, such as bisphenol A (BPA), in many canned foods, receipts, toys, and medical equipment, and some chemicals such as polybrominated diphenyl ethers and phthalates may have even increased body burden (Zota et al., 2008, 2014). Studies on these chemicals are sparse with mixed findings.

Associations between ASD and PCB are inconsistent (Kim et al., 2010; Cheslack-Postava et al., 2013; Braun et al., 2014), and the seemingly elevated risks in a pilot study (Cheslack-Postava et al., 2013) have been criticized for possible bias due to lack of adjustment for birth order (Kalkbrenner et al., 2014). Kardas et al. reported higher serum BPA concentrations in a case-control study of 48 cases of ASD and 41 controls, but no measurement of prenatal exposure was reported (Kardas et al., 2016). Braun et al. (2014) and Miodovnik et al. (2011) failed to find any association with the score of Social Responsiveness Scale (SRS), measurement of ASD traits, and maternal BPA serum or urine concentration and in their cohort studies; however, Braun et al. (2009) found that mid-pregnancy BPA concentrations were associated with an increase in externalizing problem behaviors in early childhood.

However, studies on phthalates mostly suggest an association between ASD and phthalates. Miodovnik et al. (2011) studied 137 children and found that higher phthalate metabolites in maternal urine in the third trimester were associated with a lower score on several of the SRS subscales at 7–9 years old (Miodovnik et al., 2011). Larsson et al. (2009) followed 4779 children and reported that those at 1–6 years old living in homes with polyvinyl chloride flooring (a significant source of phthalates) were 2.4 times more likely to be diagnosed with ASD (Larsson et al., 2009). Kardas et al. (2016) also reported higher serum phthalates concentrations in cases of ASD (Kardas et al., 2016). Braun et al. (2014) failed to detect an association between phthalates in maternal urine and ASD, and Phillipat et al. (Philippat et al., 2015) also failed to detect an association between house dust levels of phthalates and ASD; however, Phillipat et al. explained that the lack of association may be due to fact that the measured exposure may have only poorly reflected the actual exposure of phthalates.

## Heavy metals

There is sufficient evidence that maternal exposure to heavy metals such as lead, mercury, cadmium, and arsenic cause an increase in neurodevelopmental disorders, and restrict fetal and infant growth even at low-level exposures (De Palma et al., 2012; Ornoy et al., 2015). However, less research has been conducted on heavy metals in relation to ASD. Recently, Rossignol et al. (2014) systematically reviewed literature on environmental toxicants and summarized 40 case-control studies that compared a variety of heavy metal concentrations (i.e., lead, mercury, arsenic, cadmium, aluminum, fluoride, manganese, chromium, nickel, uranium, and tin) in blood, hair, brain, teeth, or urine in children with ASD compared to controls, as well as seven similar studies on urinary porphyrin, which is considered to have a heavy metal burden (Rossignol et al., 2014). The most studied metals were mercury (29 studies) and lead (25 studies). Although the urinary porphyrin studies collectively suggest a higher heavy metal burden among children with ASD, a recent study by Dickerson et al. (2015) found that among 2489 children the prevalence of ASD was higher when mothers were living closer to industrial facilities that released arsenic, lead, or mercury.

A meta-analysis of seven studies on the mean hair level of mercury in a total of 343 cases of ASD and 317 controls did not show any significant association between mercury and ASD (De Palma et al., 2012) and neither did a recent cohort study by van Wijngaarden et al. (2013) on 1784 children and young adults. Some studies that assessed blood have found an association between mercury and ASD (Ip et al., 2004; Desoto and Hitlan, 2007; Geier et al., 2010), whereas others have not (Hertz-Picciotto et al., 2010; Stamova et al., 2011; Albizzati et al., 2012; Adams et al., 2013; Rahbar et al., 2013). However, the lack of adjusting for strong protective factors such as fish oil that are ingested concomitantly in many of the studies (Karagas et al., 2012) and the possible conflict of interest with industries (Kern et al., 2015) may be masking existing associations, as studies on air-borne mercury consistently report an association between mercury exposure and ASD (Windham et al., 2006; Roberts et al., 2013).

## Micronutrients

Micronutrients are essential for neurogenesis and the development of the neuro-network (Curtis and Patel, 2008). Lower levels of magnesium (Strambi et al., 2006), zinc (Adams and Vogelaar, 2005), selenium (Adams and Vogelaar, 2005), vitamin A (Adams and Vogelaar, 2005), vitamin B complex (Adams and Vogelaar, 2005; Pineles et al., 2010), vitamin D (Adams and Vogelaar, 2005; Gong Z. L. et al., 2014; Kocovska et al., 2014), vitamin E (Adams and Vogelaar, 2005), and carnitine (Filipek et al., 2004) in blood, hair, or other tissue among children with ASD have been reported. Further, the association between a deficiency of micronutrients during pregnancy, such as folic acid (Schmidt et al., 2011, 2012; Suren et al., 2013) and vitamin D (Cannell, 2008; Grant and Soles, 2009), have been reported as a risk for offspring developing ASD.

These previous studies advanced to intervention studies to confirm the causality or possibility of using nutrients to treat ASD. Several studies have reported that nutritional intervention showed a trend toward improvement in patients with ASD. For example, a double-blind study on 20 children (age 3–8 years) with ASD who took a broad-based multi-vitamin and mineral supplement suggested the possible benefit of improving general behavior and receptive language, although this finding was not significant (Adams and Holloway, 2004). Another double-blind study reported that supplementing L-carnosine to children (age 3–12 years) with ASD showed statistically significant improvements in the symptoms on ASD (Chez et al., 2002). In another study, it was also reported that oral magnesium and vitamin B6 supplements led to improvements in social interactions, communication, stereotyped restricted behavior, and abnormal/delayed functioning among children (age 1–10 years) with ASD (Mousain-Bosc et al., 2006).

Several studies have reported the association between gender and ASD in the relationship with micronutrients. For example, a study conducted in the Faroe Islands (Kocovska et al., 2014) noted the trend for ASD males having lower levels of vitamin D and 25(OH)D3. Similarly, another study suggested that the differenteial effects of estrogen and testosterone on vitamin D metabolism might explain the gender difference of ASD (Cannell, 2008).

## Fatty acids

As neural development requires essential fatty acids, particularly long-chain omega-3 fatty acids during critical growth periods, and inflammation may be associated with ASD (Ornoy et al., 2015), the fatty acid level may play an important role in the development of ASD. Several studies have shown that both red blood cell and plasma fatty acid composition among cases of ASD differ from those of non-ASD people. Specifically, the levels of omega-3 fatty acids (Vancassel et al., 2001; Bell et al., 2004; Brigandi et al., 2015), docosahexaenoic acid (DHA) (Meguid et al., 2008; Wiest et al., 2009; El-Ansary et al., 2011; Al-Farsi et al., 2013; Brigandi et al., 2015), and arachidonic acid (AA) (Meguid et al., 2008; El-Ansary et al., 2011; Brigandi et al., 2015; Yui et al., 2016) were significantly lower in the red blood cell or plasma of cases of ASD compared to controls, although some studies did not support these claims (Bu et al., 2006; Bell et al., 2010). To date, only one study has examined maternal fatty acid intake during pregnancy is association with ASD (Lyall et al., 2013). Women with higher intake of polyunsaturated fatty acids (PUFA) before and during pregnancy had a reduced risk of having a child with ASD than those with lower PUFA intake. Analysis on specific PUFAs showed that women in the highest quartile of intake of omega-6 fatty acids had a 34% reduction in the risk of having a child with ASD compared with those in the lowest quartile, with similar results for linoleic acid intake. In concern with omega-3 fatty acids, women with very low intakes (i.e., the lowest 5% of the distribution) of had a significantly increased risk of having a child with ASD compared with those in the middle 90% of the distribution.

Reports on the benefits of fatty acid supplementation in children with ASD are inconclusive. Recently, Mankad et al. (2015) conducted a randomized controlled 6-month trial of 1.5 g/day of omega-3 fatty acids or a placebo in 38 children aged 2–5 years with ASD, and found no evidence for the efficacy of omega-3 fatty acids on improving core symptoms (Mankad et al., 2015). However, Ooi et al. (2015) conducted a 12-week open-label study of 1 g/day of omega-3 fatty acids in 41 children aged 7–18 years with ASD, and found significant improvements in the core symptoms and attention problems (Ooi et al., 2015). Yui et al. (2012) conducted a randomized controlled 16-week trial of AA and DHA supplementation or a placebo in 13 individuals aged 6–28 years with autism, and found significant improvements in social withdrawal and communication (Yui et al., 2012). These studies were relatively small, thus the findings may be by coincidental so a further larger randomized controlled trial is needed.

## Parental obesity

Maternal obesity can be associated with having offspring with ASD due to the accumulation of the aforementioned chemicals, or it can serve as a proxy of poor nutrition (Dodds et al., 2011; Kawicka and Regulska-Ilow, 2013; Ornoy et al., 2015). According to a Swedish cohort study of 333,057 participants, which included 6420 individuals with ASD, maternal overweight or obesity evaluated at the first antenatal visit was associated with having an offspring with ASD (Gardner et al., 2015). However, the association between an elevated maternal body mass index and the risk of ASD was not clear in matched sibling analyses.

In a population-based prospective cohort study of 92,909 children (age 4–13 years), Suren et al. (2014) investigated the association between ASD and paternal obesity recorded in the questionnaires answered by the fathers. They found that paternal obesity was associated with an increasing risk of ASD (adjusted OR: 1.73, 95% CI: 1.07, 2.82), whereas maternal obesity showed only a weak association with ASD (Suren et al., 2014).

## Summary and future directions

In summary, several chemical exposures such as air pollution (e.g., PM 2.5), pesticides, BPA, phthalates, mercury or lead, and nutrition deficiencies such as folic acid, vitamin D, or fatty acid are possibly associated with the onset of ASD, whereas other traditional risk factors such as smoke/tobacco, alcohol, or PCB are less likely to be associated with ASD. Apparently, no single environmental factor can explain the development of ASD, suggesting that upstream environmental factors such as socioeconomic status need to be considered as risk factors for ASD, which have not been as rigorously investigated (Fujiwara, 2014). Further, few studies have investigated the accumulative or synergistic effect of the different chemical exposures and nutrition deficiencies simultaneously. The impact of multiple exposures to chemicals and nutrient deficiencies, which are suggestive of association with ASD, need to be studied together to assess whether effect is additive or multiplicative. Moreover, not all children exposed to these chemicals or nutrients may have risk of developing ASD, suggesting that some genetic polymorphism related to ASD, such as *CD38* (Higashida et al., 2012), may have an interaction effect with these environmental exposures during the onset of ASD, as studied in the exposure of heavy mental and genetic polymorphism related to metabolism (Rossignol et al., 2014). Moreover, few chemical or nutritional exposures were investigated to elucidate the mechanism of gender difference of ASD prevalence. These uncovered topics need to be investigated in future research.

## Author contributions

TF conceived the review focus, conducted literature review, summarized, and finalized the manuscript. NM, YH, MS, and YT reviewed literature, wrote first draft, and finalized the manuscript. All authors approved final version of manuscript.

## Funding

This study was supported from Japan Agency for Medical Research Development (16gk0110001h0003).

### Conflict of interest statement

The authors declare that the research was conducted in the absence of any commercial or financial relationships that could be construed as a potential conflict of interest.
